# Humoral response in experimental autoimmune encephalomyelitis targets neural precursor cells in the central nervous system of naive rodents

**DOI:** 10.1186/s12974-017-0995-2

**Published:** 2017-11-21

**Authors:** Evangelia Kesidou, Olga Touloumi, Roza Lagoudaki, Evangelia Nousiopoulou, Paschalis Theotokis, Kyriaki-Nepheli Poulatsidou, Marina Boziki, Evangelia Kofidou, Nickoleta Delivanoglou, Fani Minti, Georgios Hadjigeorgiou, Nikolaos Grigoriadis, Constantina Simeonidou

**Affiliations:** 1 Laboratory of Experimental Neurology and Neuroimmunology, 2nd Department of Neurology, AHEPA University Hospital, Aristotle University of Thessaloniki, Thessaloniki, Greece; 2 Laboratory of Experimental Physiology, Faculty of Medicine, Aristotle University of Thessaloniki, Thessaloniki, Greece; 3 Department of Neurology, Faculty of Medicine, University of Thessaly, Larissa, Greece

**Keywords:** Experimental autoimmune encephalomyelitis, Neural precursor cells, Autoantibody, Subventricular zone, Remyelination, Apoptosis

## Abstract

**Background:**

Neural precursor cells (NPCs) located in the subventricular zone (SVZ), a well-defined NPC niche, play a crucial role in central nervous system (CNS) homeostasis. Moreover, NPCs are involved in the endogenous reparative process both in multiple sclerosis (MS) and experimental autoimmune encephalomyelitis (EAE). However, the possibility that NPCs may be vulnerable to immune-related components may not be ruled out. Therefore, we investigated the potential affinity of myelin oligodendrocyte glycoprotein (MOG)-induced humoral response(s) to NPCs.

**Methods:**

MOG_35–55_-EAE was induced in C57BL/6 mice; blood-sampling was performed on days 17–21 (acute phase) along with a naive group and corresponding antisera (AS) were collected (EAE-AS, NAIVE-AS). The presence of anti-CNS autoantibodies was examined with western blotting. Furthermore, using the collected antisera and anti-MOG antibody (as positive control), immunohistochemistry and double immunofluorescence were implemented on normal neonatal, postnatal, and adult mouse brain sections. Targeted NPCs were identified with confocal microscopy. In vitro immunoreactivity assessment on NPCs challenged with autoantibodies was evaluated for apoptotic/autophagic activity.

**Results:**

Western blotting verified the existence of autoantibodies in EAE mice and demonstrated bands corresponding to yet unidentified NPC surface epitopes. A dominant selective binding of EAE-AS in the subventricular zone in all age groups compared to NAIVE-AS (*p* < 0.001) was observed. Additionally, anti-BrdU^+^/EAE-AS^+^ colocalization was significantly higher than anti-BrdU^+^/anti-MOG^+^, a finding suggesting that the EAE humoral response colocalized with NPCs(BrdU^+^), cells that do not express MOG. Well-established NPC markers (Nestin, m-Musashi-1, Sox2, DCX, GFAP, NG2) were used to identify the distinct cell types which exhibited selective binding with EAE-AS. The findings verified that EAE-AS exerts cross-reactivity with NPCs which varies throughout the neonatal to adult stage, with a preference to cells of early developmental stages. Finally, increased expressions of Caspase 3 and Beclin 1 on NPCs were detected.

**Conclusion:**

We provide evidence for the first time that MOG_35–55_ EAE induces production of antibodies with affinity to SVZ of naive mice in three different age groups. These autoantibodies target lineage-specific NPCs as brain develops and have the potential to trigger apoptotic pathways. Thus, our findings provide indication that cross-talk between immunity and NPCs may lead to functional alteration of NPCs regarding their viability and potentially oligodendrogenesis and effective remyelination.

**Electronic supplementary material:**

The online version of this article (10.1186/s12974-017-0995-2) contains supplementary material, which is available to authorized users.

## Background

Over the last decades, scientists have focused on understanding the ambiguous and multifactorial concept of autoimmunity. Autoimmunity is defined as the improper recognition of physiological proteins by immune cells and activation of immune mechanisms (cellular and humoral immunity) aimed at their damage. The causes that turn the organism’s defense mechanism against normal structures are not yet clarified. Multiple sclerosis (MS) is a complex autoimmune disease which involves T cell mediation. However, it has been shown that B cells also contribute to MS pathogenesis. The detection of oligoclonal immunoglobulins IgM and IgG in the cerebrospinal fluid (CSF) of MS patients is evidence of the existence of activated B cell clones in the CSF [1, 2].

The question regarding the identity of the antigenic target and the specific immunoglobulins generated in MS remains unanswered. One potential target of the immune response, apart from the myelin sheath, seems to be cells that can restore damage of the central nervous system (CNS), namely, the neural precursor cells (NPCs). NPCs reside in specific germinal regions, the subventricular zone (SVZ) and the subgranular zone (SGZ) of the hippocampal dentate gyrus. SVZ is characterized as a defined stem-cell niche and contributes to gliogenesis and neurogenesis [3, 4]. Both in MS and EAE, endogenous NPCs differentiate mainly into glial cells, particularly into oligodendrocyte precursor cells (OPCs) and migrate in demyelinating areas of the white matter [5]. The inflammatory microenvironment interferes with the differentiation and maturation of NPCs resulting in defective remyelination. A therapeutic approach that has been proposed for demyelination is restoration through NPC transplantation [6]. However, controlling the differentiation process is difficult, since it depends on the existing microenvironment. Although NPCs exhibit chemotaxis to demyelination foci, they are unable to migrate towards multiple scattered areas. NPC growth potential and immunomodulatory exertion also need to be taken into account [7, 8].

Several animal models attempt to mimic the disease, but the variability of MS characteristics complicates understanding of MS pathophysiological features. One of the most common and reliable animal models is experimental autoimmune encephalomyelitis (EAE) induced by active immunization of mice with the immunogenic peptide epitope MOG_35–55_ of myelin oligodendrocyte glycoprotein (MOG). MOG is expressed only in the CNS on the outer surface of the myelin sheath and on oligodendrocyte processes. There are no detectable levels in the periphery, as opposed to other myelin proteins, myelin basic protein (MBP), and myelin proteolipid protein (PLP), which are also used in experimental animal models of MS [9]. It is believed that the soluble form of MOG is cleared directly by endogenous immune mechanisms within the CNS. Additionally, it has been found that autoantibodies against MOG mediate demyelination in vitro [10] and in EAE [11], acting against extracellular epitopes of MOG [12]. However, clinical relevance of anti-MOG antibodies in MS has been disputed. Nonetheless, humoral immunity and B cell implication in MS are still under investigation [13].

To our knowledge, studies of humoral responses in EAE and autoantibody potential to target CNS progenitor cells (NPCs) in the SVZ have not been conducted previously. In the present study, we examined whether activation of the immune system against MOG_35–55_ may target SVZ progenitor cells. Here, we provide evidence that rodents after MOG_35–55_ immunization produce an antibody repertoire which exhibits cross-reactivity with NPC surface. This evidence may indicate the effect of antibodies on NPC function leading to decreased oligodendrogenesis and/or inefficient remyelination.

## Methods

### Animal handling

Female C57BL/6 mice, 8–10 weeks old, were purchased from the Hellenic Pasteur Institute (Athens, Greece) and housed in the animal facility of AHEPA University Hospital (Thessaloniki, Greece). The required approval from the Veterinary Directorate of Thessaloniki (25951/143) was obtained. Animals were fed an ordinary diet and were given water without antibiotics ad libitum. Animal handling was conducted according to the National Ministry of Health (Athens, Greece) guidelines, the Greek Regulations, and the local ethics committee.

### Experimental groups, EAE induction, and evaluation

The current project consisted of five experimental groups. The EAE group (*n* = 10), CFA (complete Freund’s adjuvant) group (*n* = 7), naive adults (3 months old, *n* = 12), naive postnates-P17 (17 days old, *n* = 7), and naive neonates-P3 (3 days old, *n* = 7). Five mice (*n* = 5) of the naive adult group were used as negative control. In the EAE group, ΜΟG_35–55_ EAE was induced using standard protocol [14, 15] (details in Additional file 1). Serum was collected from the mice of the three experimental groups: EAE group (*n* = 10), CFA group (*n* = 7), and naive adult group (*n* = 5). Naive adults (*n* = 7), naive postnates (*n* = 7), and naive neonates (*n* = 7) were used for tissue collection.

### Tissue and serum collection

The method of euthanization was selected depending on the age of the rodent. Specifically, naive adult animals were perfused transcardially with 4% paraformaldehyde (PFA) after sedation with ether inhalation, whereas organs from postnates and neonates were directly isolated and immersed in 4% PFA (PanReac AppliChem GmbH, Germany) after ether inhalation and verification of death by decapitation. Post-fixation for approximately 20 h, sectioning and paraffin embedding followed. On days 17–21 (acute phase of EAE) blood sampling from orbital cavity under isoflurane anesthesia was performed from mice of EAE, CFA, and naive adult groups, in order to obtain the antisera (AS); EAE-AS, CFA-AS, and NAIVE-AS, correspondingly. Collected antisera were stored at − 80 °C until further use.

### BrdU administration

Naive mice (7 adults, 7 postnates, and 7 neonates) received three doses of bromodeoxyuridine (5-bromo-2′-deoxyuridine, BrdU; Sigma-Aldrich GmbH, Germany) i.p., within a period of 6 h and were euthanized 1 h after the last injection. The regular BrdU dose was 80 mg/kg of body weight dissolved in PBS, pH 7.2–7.4. BrdU incorporates into DNA of dividing cells thus in naive mouse brain it labels neural stem cells [16].

### Cell culture protocol

NPCs were cultured from newborn C57BL/6 J mice as previously described [17, 18] (details in Additional file 1). NPCs were cultured for 7 days under constant conditions of 10% CO_2_ and 37 °C, with the presence of 10 μg/ml basic fibroblast growth factor (bFGF/Peprotech, Rocky Hill, New Jersey, USA) and 10 μg/ml epidermal growth factor (EGF/Peprotech, Rocky Hill, New Jersey, USA). After this period, neurospheres were harvested for protein isolation and immunocytochemistry assay.

MOG-transduced EL4 cells (EL4-MOG) were kindly provided by Dr. Gurumoorthy Krishnamoorthy, Research group neuroinflammation and mucosal immunology, Max Planck Institute of Biochemistry (Martinsried, Germany), and cultured according to standard protocol [19] (details in Additional file 1).

Murine Neuro2a (N2a) cell line was kindly provided by Prof. T. Sklaviadis, Pharmacy department of A.U.TH (Thessaloniki, Greece), and cultured according to standard protocol [20].

### IgG purification assay

Melon™ gel IgG spin purification kit (Thermo Fischer Scientific, Haverhill, MA USA) was implemented as described in manufacturers’ datasheet. EAE-AS, CFA-AS, and NAIVE-AS were used in 1:10 dilution in Melon gel purification buffer, eluted from the column and incubated with the different antisera for 5 min. Pure IgG from each antiserum was collected in the flow-through fraction of the microcentrifuge tube, through Melon gel resin.

### XTT viability assay and in vitro comparative immunoreactivity assessment

TACS® XTT cell viability assay (R&D Systems, Minneapolis, Minnesota, USA) was used according to the manufacturer’s instructions. NPCs were seeded on a 96-well microplate (Corning, New York, USA) at a density of 10^5^ cells/100 μl and incubated for 48 h with purified IgG from EAE-AS and unpurified EAE-AS, as well as with purified IgG from NAIVE-AS and unpurified NAIVE-AS (negative control) at varying concentrations ranging from 0.1 to 10 μg/ml as determined by spectrophotometrical quantification using BCA protein quantification kit (Abcam, Cambridge, UK). XTT reagent was added for 6 h and absorbance at 490 nm was measured by microplate spectrophotometer reader (Molecular Devices, Sunnyvale, California, USA). Relative cell viability (%) of NPCs exposed to purified IgG from EAE-AS and unpurified EAE-AS was expressed as a percentage relative to cells treated with purified IgG from NAIVE-AS and unpurified NAIVE-AS, respectively.

In order to assess neurosphere immunoreactivity, 5000 neurospheres/flask and N2a cells, (15,000 cells/culture dish), serving as a negative control, were challenged with the aforementioned purified IgG from antisera (EAE-AS, CFA-AS, and NAIVE-AS) in final concentration of 100 μg/ml. Neurospheres and N2a cells were incubated without growth factors for 48 h and harvested for immunocytochemistry assay.

### Western blotting

Total protein lysate was isolated from NPC neurospheres and from naive adult mouse spinal cord (details in Additional file 1).Twenty to thirty micrograms of lysates were diluted in loading buffer and were subjected to SDS-PAGE, transferred onto PVDF membrane (Macherey-Nagel, GmbH&Co, Germany), cut into 2-mm strips, and incubated with EAE-AS, CFA-AS, NAIVE-AS (negative control), and goat anti-MOG (polyclonal antibody, positive control, R&D Systems, Minneapolis, Minnesota, USA) overnight at 4 °C. Membranes were washed with PBS supplemented with 0.1%Tween 20 (PBST) and incubated with HRP-conjugated anti-mouse IgG (Cell Signaling Technology, Leiden, The Netherlands) for 1 h in room temperature. Immunoreactivity was visualized by enhanced chemiluminescence (ECL, GenScript, New Jersey, USA). Membranes were stripped and incubated with anti-actin antibody (Cell Signaling Technology, Leiden, The Netherlands) to verify equal loading of samples onto the gel.

Recombinant MOG (R&D Systems, Minneapolis, Minnesota, USA) was blotted and it was detected by anti-MOG and EAE-AS and served as a positive control for the existence of anti-MOG immunoglobulins within EAE-AS.

### Immunohistochemistry (IHC) and immunocytochemistry (ICC)

For IHC, brain paraffin sections were deparaffinized, hydrated, rinsed, and then blocked with hydrogen peroxide in methanol. Incubation of sections with blocking buffer (10% fetal bovine serum, FBS; Merck Millipore, Billerica, MA, USA) that excludes binding to non-specific antigenic sites followed. Due to the fact that mouse tissue and mouse antisera were used, an extra incubation with goat anti-mouse Fab fragment (Jackson ImmunoResearch, WestGrove, PA, USA) was applied to block endogenous immunoglobulins and to offer an additional prevention from non-specific binding. EAE-AS, CFA-AS, and NAIVE-AS (as primary antibodies) were added in the appropriate concentration (1:1000, after titration test) overnight. The next day, biotinylated horse anti-mouse IgG (Thermo Fischer Scientific, Haverhill, MA, USA) was added, followed by avidin (Sigma-Aldrich GmbH, Germany) conjugated to peroxidase, in order to make the avidin-biotin-peroxidase complex. Every staining was evaluated with incubation of sections without primary antibody in order to determine any background signal resulting from the secondary antibody. Positive cells were visualized by 3,3′ diaminobenzidine-tetra-hydrochloride (DAB; Fluka, Honeywell, Mexico City, Mexico) and sections were counterstained with hematoxylin (Merck Millipore, Billerica, MA, USA). For the ICC, suitable glass coverslips in 35-mm culture dishes were coated with 10 μg/ml poly-D-lysine (Sigma-Aldrich GmbH, Germany) and 10 μg/ml recombinant human fibronectin (Sigma-Aldrich GmbH, Germany). Neurospheres (250–300 spheres/culture dish) were incubated for 4 h at 10% CO_2_ and 37 °C in order to allow cells to adhere. Cells were fixed with 4% PFA solution, washed with PBS, PBST, and 7 min incubation with PBS and 0.3% Triton-X 100 solution (Sigma-Aldrich GmbH, Germany) followed. Blocking buffer, antisera staining, secondary antibody (horse anti-mouse IgG), and avidin solutions were added as in IHC assay. DAB and hematoxylin were used for visualizing and nuclei staining. Neurospheres and N2a cells challenged with antisera were harvested, placed into coated coverslips, and fixed as mentioned above. These cells were stained with anti-Caspase 3 (R&D Systems, Minneapolis, Minnesota, USA) and anti-Beclin 1 (Santa Cruz Biotechnology, Dallas, Texas, USA); appropriate secondary antibody goat anti-rabbit IgG (Vector Laboratories, Burlingane, California, USA) were added and visualized with DAB staining. MOG-transduced EL4 cells, (approximately 25,000 cells/200 μl) were added into coated coverslips and fixed on 4% PFA. Cells were stained with antisera (EAE-AS, CFA-AS, and NAIVE-AS) and anti-MOG, horse anti-mouse IgG, and rabbit anti-goat IgG (Thermo Fischer Scientific, Haverhill, MA, USA), respectively, as secondary antibodies and chromogenic DAB.

### Double (dIF) and triple (tIF) immunofluorescence staining

In order to determine whether the antisera exhibited binding with NPCs in brain sections, double immunofluorescence was used. Identification of EAE-AS positive NPCs (BrdU^+^) was performed using triple immunofluorescence. Brain sections from naive animals, injected with BrdU, were used for dIF and tIF staining. Briefly, the protocol procedure included deparaffinization, hydration, and denaturation of nuclear DNA using a 2 N hydrochloric acid (HCL) solution (Sigma-Aldrich GmbH, Germany). Neutralization with borax 0.1 M solution (Sigma-Aldrich GmbH, Germany), blocking buffer, and goat anti-mouse Fab fragment incubation followed. Primary antibodies were applied overnight with the following combinations: for dIF: rat anti-BrdU (Abcam, Cambridge, UK)/NAIVE-AS, rat anti-BrdU/EAE-AS, rat anti-BrdU/goat anti-MOG, rat anti-BrdU/mouse anti-Nestin (Cell Signaling Technology, Leiden, The Netherlands), rat anti-BrdU/mouse anti-Musashi-1 (Santa Cruz Biotechnology, Dallas, Texas, USA). For tIF: rat anti-BrdU (Abcam, Cambridge, UK)/EAE-AS with the following markers: goat anti-SOX-2 (Santa Cruz Biotechnology, Dallas, Texas, USA), goat anti-DCX (Santa Cruz Biotechnology, Dallas, Texas, USA), and rabbit anti-GFAP (DAKO, Agilent Technologies, Santa Clara, California, USA). The following secondary antibodies: goat anti-rat (Biotium 555, Biotium, Fremont, California, USA), chicken anti-mouse (Biotium 488, Biotium, Fremont, California, USA), chicken anti-goat (Biotium 633, Biotium, Fremont, California, USA), chicken anti-rabbit (Alexa Fluor 647, Thermo Fischer Scientific, Haverhill, MA, USA) were used appropriately. Sections were incubated without primary antibody in order to determine any background signal deriving from the secondary antibody. DAPI mounting medium (Biotium, Fremont, California, USA) was used for nuclei counterstaining.

### Neuropathological evaluation

Stained sections were observed under optical, fluorescent microscope (Zeiss Axioplan II) and under confocal microscope (Nikon Eclipse TE2000U). Fiji software was used in order to quantify immunoreactive cells and measure integrated density of DAB and fluorescence. Twelve microscopic fields (×20 magnification) containing neurospheres (diameter 50–150 μm) were observed and positive cells migrating from the sphere (30–50 μm from the edge) were measured and presented as percentage positive cells/total cells. For counts of double- and triple-labeled cells, results are expressed as positive cells/mm^2^ or percentage co-expression. SVZ sections were examined at ×40 magnification. Tissue sections and cells were evaluated blinded by two individual observers.

### Statistical analysis

Data were analyzed using GraphPad Prism 5 and SPSS 18.0 software. Kolmogorov-Smirnov and Shapiro-Wilk tests were used to assess the normality of the distributions. For parametric data, differences among experimental groups were evaluated by one-way analysis of variance and post hoc Bonferroni’s test. The Kruskall-Wallis and Dunn’s post hoc were used to evaluate non-parametric data. Regarding the evaluation of differences between two experimental groups, unpaired *t* test was used to evaluate parametric data and Mann-Whitney test was used to evaluate non-parametric data. Results are presented as mean ± SEM and differences were considered statistically significant when *p* < 0.05 (*p < 0.05, **p < 0.01, ***p < 0.001).

## Results

### Effect of purified IgG from EAE-AS, unpurified EAE-AS on neurosphere viability

**Fig. 1:**
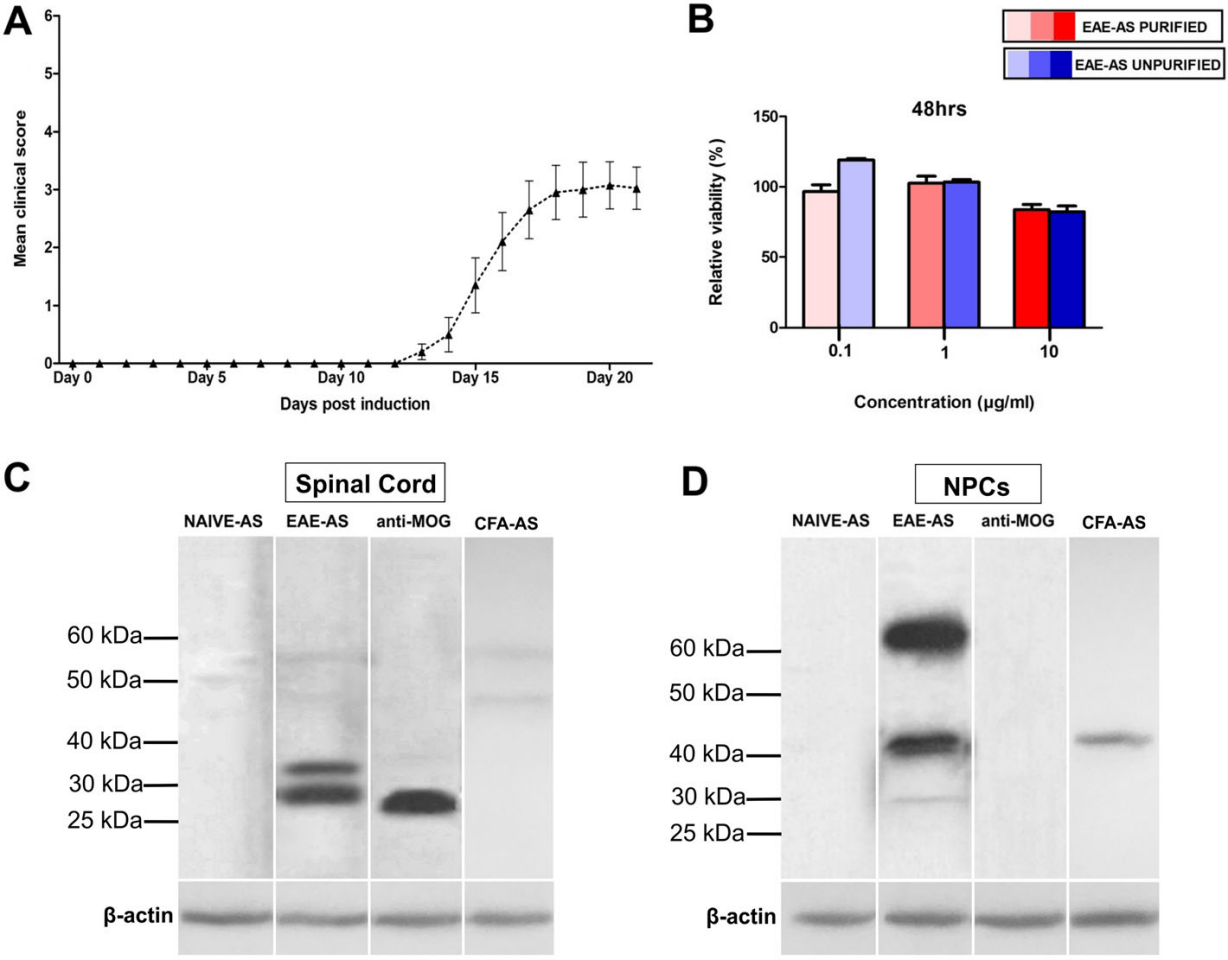
MOG_35–55_-EAE induction elicits a humoral response directed towards the spinal cord and NPCs. **a** Mean clinical score of all animals during EAE course. Error bars show the standard statistical error of the mean (SEM). **b** XTT assay indicated the relative NPC viability % of NPCs treated with purified IgG from EAE-AS and unpurified EAE-AS compared with NPCs treated with NAIVE-AS (control), in three different concentrations (0.1, 1, and 10 μg/ml). Data are presented as relative viability percentage (%) as mean ± SEM. Western blot of various antisera from animals immunized with MOG (EAE-AS) yielded one band approximately at 30 kDa on spinal cord substrate (**c**) and bands at above 60 kDa, above 40 kDa, and around 30 kDa on NPC substrate (**d**). Lane probed with EAE-AS demonstrates a representative antiserum. Anti-MOG antibody and anti-actin-loading control were also used

Autoantibody response against MOG_35–55_-EAE was determined when the maximum score of EAE occurred (acute phase, day 17–21; Fig. 1a). In order to examine whether IgGs from EAE-AS could affect NPC viability, IgG isolation from EAE-AS was performed with Melon™ Gel IgG Spin Purification Kit. Purified IgG from EAE-AS and unpurified EAE-AS and purified IgG from NAIVE-AS and unpurified NAIVE-AS (control) were added to NPCs in different concentrations (0.1, 1, and 10 μg/ml). XTT assay confirmed that NPCs remain viable in the presence of purified IgG from EAE-AS and unpurified EAE-AS. Furthermore, purified IgG from EAE-AS and unpurified EAE-AS exert the same effect on NPC viability (not statistically significant; Fig. 1b). Additionally, western blot of NPC lysate demonstrated bands of same molecular weight when purified IgG from EAE-AS and unpurified EAE-AS were used (Additional file 2: Figure S1A).

### Immunoreactivity of EAE-AS on spinal cord and NPC lysate generates a specific response

In order to explore whether immunization with MOG elicits specific immune response, (autoantibodies against MOG) western blotting was performed on total naive spinal cord lysate. EAE-AS showed immunostaining of the expected band at around 30 kDa, which corresponds to MOG protein [21], also verified by anti-MOG, a commercially available antibody which served as positive control (distinct band at 28–30 kDa). Reactivity of NAIVE-AS on spinal cord lysate was not observed (Fig. 1c). In total NPC lysate, EAE-AS reacted with four specific bands (one above 60 kDa, two bands above 40 kDa, and one band around 30 kDa). Three bands could not be attributed to CFA, since when CFA-AS was used, only one band at around 45 kDa was observed. No similar reaction was found when anti-MOG antibody and NAIVE-AS were used (Fig. 1d). Moreover, the existence of anti-MOG-immunoglobulins within EAE-AS was confirmed using recombinant MOG as a substrate. One expected band corresponding to MOG (22–24 kDa) was detected when anti-MOG and EAE-AS were used (Additional file 2: Figure S1B). Additionally, MOG-transduced EL4 cells (positive control as they express only MOG protein) were also positively stained with EAE-AS and anti-MOG antibody (Additional file 2: Figure S1C).

### Affinity of EAE-AS and NAIVE-AS on dissociated neurospheres and their specific binding site(s) in the SVZ

**Fig. 2:**
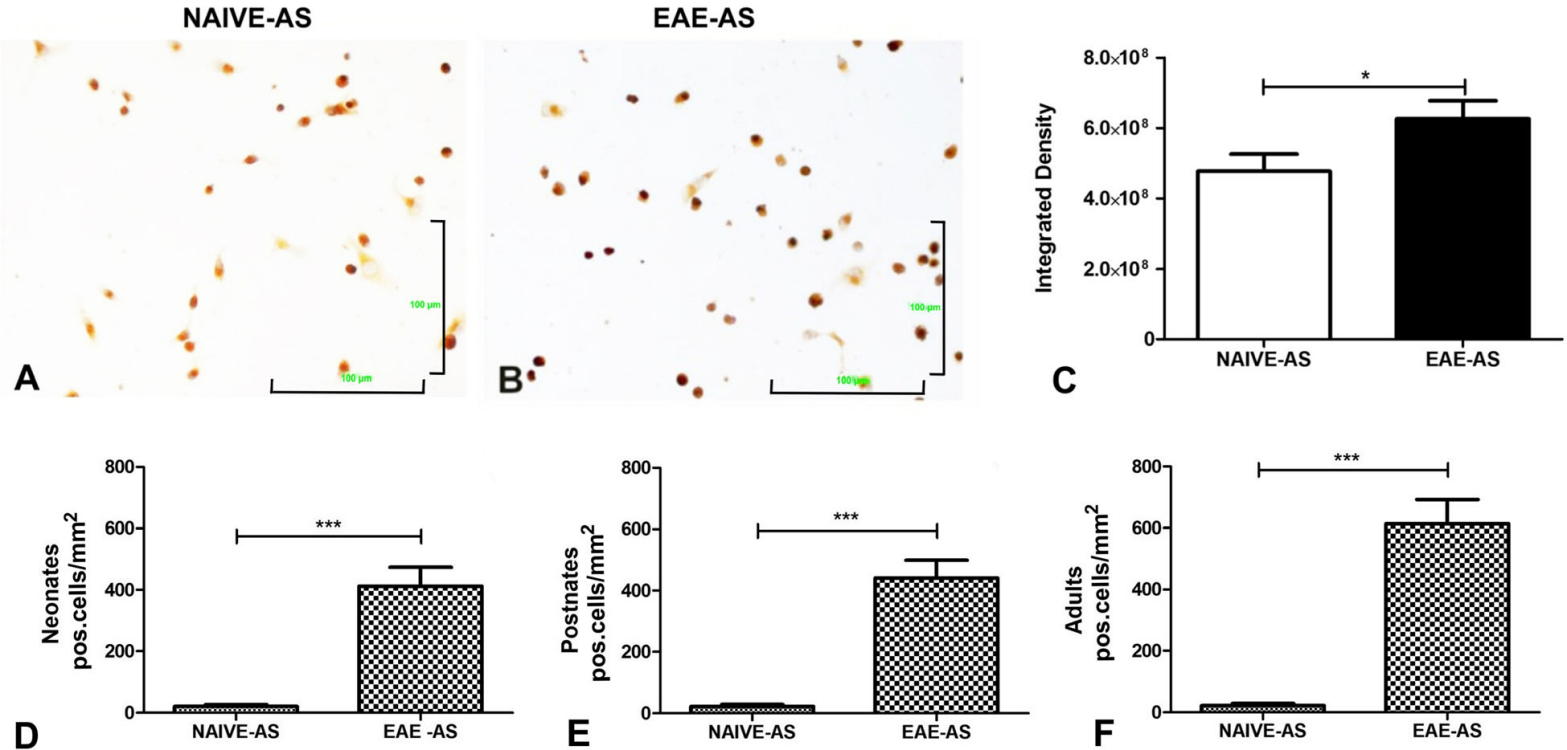
Antisera cross-reactivity to NPCs and increased selective binding to SVZ. **a** NAIVE-AS and **b** EAE-AS binding (DAB-brown staining) on dissociated neurospheres. Magnification=×40, Scale=100 μm. **c** Graphs demonstrate the integrated density of positive cells. **d**–**f** Bars represent DAB positive cells/mm^2^ in SVZ and periventricular area from three different developmental stages of normal mouse brain stained with NAIVE-AS and EAE-AS. Error bars indicate SEM (* *p* < 0.05, *** *p* < 0.001)

In order to examine the immunoreactivity of antisera on cultured dissociated NPCs in vitro, we stained NPCs with NAIVE-AS and EAE-AS. ICC data showed a higher integrated density of EAE-AS (6.266e + 008 ± 5.103e + 007), compared to NAIVE-AS (4.772e + 008 ± 4.912e + 007), *p* < 0.05 (Fig. 2a–c). To further explore and identify antiserum specific binding site(s) in the CNS, the same antisera were used as primary antibodies for staining normal mouse brain sections from mice of three different age groups. A plethora of EAE-AS positive cells were observed opposed to NAIVE-AS, specifically in neonates-P3 412.5 ± 61.30 versus 21.13 ± 6.081, postnates-P17: 441.0 ± 57.73 versus 22.75 ± 6.026, adults-3 months 613.9 ± 78.75 versus 22.00 ± 7.094, respectively. EAE-AS also exhibited binding along axons. However, the precise identification of molecules, beyond MOG, that may be recognized by the EAE-AS remains to be clarified. Conclusively, we observed in neonates, postnates, and adults a cell population, located in subventricular zone and periventricularly, that was recognized by EAE-AS and not by naive-AS (*p* < 0.001) (Fig. 2d–f).

### Detection of BrdU^+^/EAE-AS^+^ and BrdU^+^/anti-MOG^+^ in SVZ

**Fig. 3:**
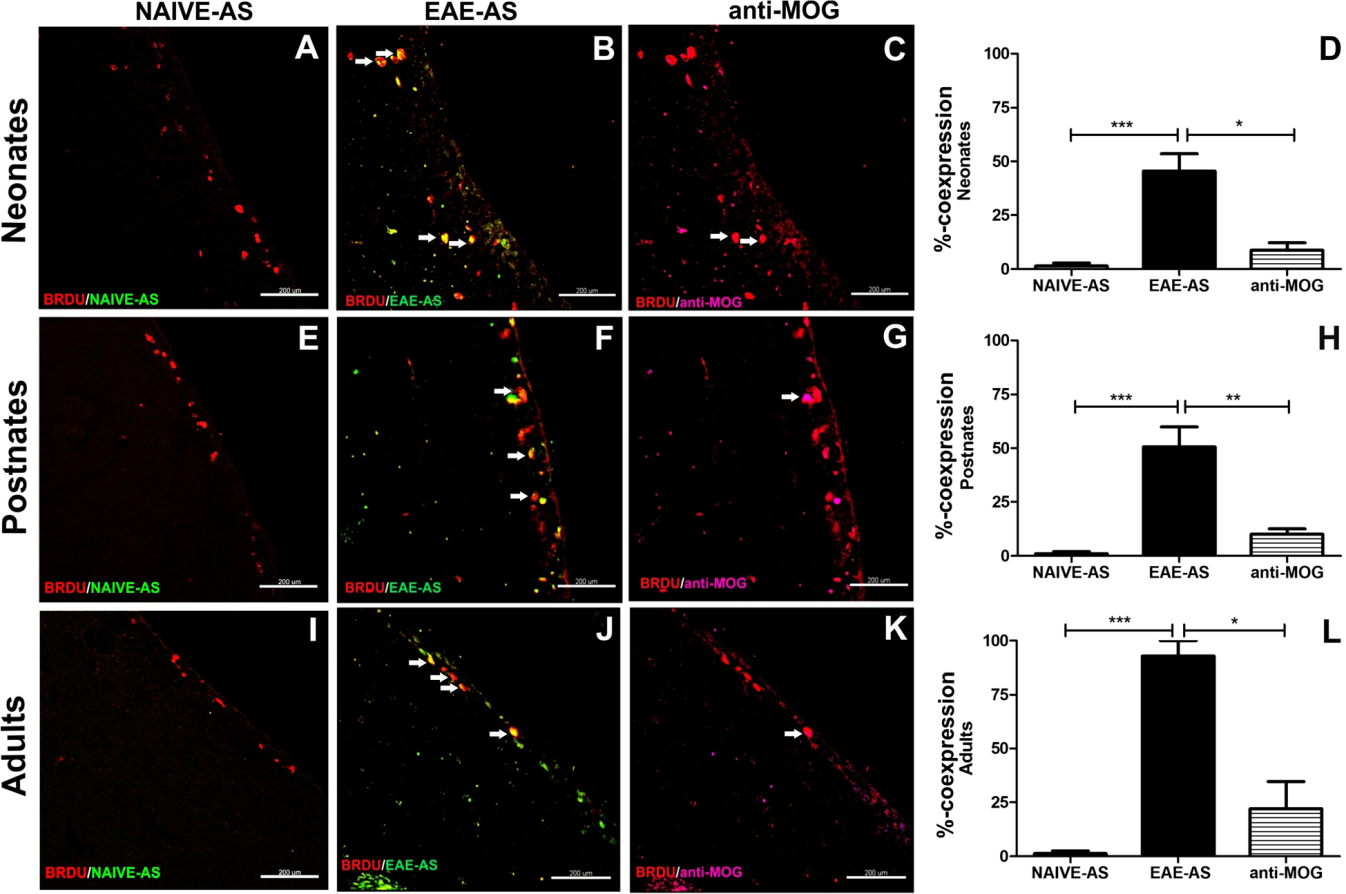
NAIVE-AS, EAE-AS, and anti-MOG binding on BrdU^+^ cells in normal mouse brain in three age groups. **a**–**c** SVZ sections of neonates, **e**–**g** postnates, and **i**, **j**, **k** adults double-stained with anti-BrdU and NAIVE-AS, EAE-AS, and anti-MOG, respectively. Arrows denote the double positive cells for BrdU^+^/EAE-AS^+^ and BrdU^+^/anti-MOG^+^. Scale bars=200 μm. **d**–**l** Graphs represent the corresponding anti-BrdU co-expression % with NAIVE-AS, EAE-AS, and anti-MOG. BrdU^+^/EAE-AS^+^ cell populations are widespread in three age groups versus BrdU^+^/anti-MOG^+^, especially in postnates (* *p* < 0.05, ** *p* < 0.01, *** *p* < 0.001)

Increased EAE-AS immunoreactivity to neonatal, postnatal, and adult SVZ led us to explore whether MOG-induced antiserum (EAE-AS) colocalized with NPCs (BrdU^+^ cells). To confirm this concept, dIF was conducted with double staining of normal SVZ sections of all age groups with anti-BrdU/NAIVE-AS (Fig. 3a–i), anti-BrdU/EAE-AS (Fig. 3b–j), and anti-BrdU/anti-MOG (Fig. 3c–k), respectively. NAIVE-AS was used as negative control and anti-MOG antibody as positive control. Our data revealed that EAE-AS has a significantly higher binding to BrdU^+^ cells parallel to anti-MOG (%co-expression): neonates, 45.43 ± 8.112% versus 9.490 ± 3.631% (*p* < 0.001; Fig. 3d); postnates, 50.64 ± 9.207% versus 10.13 ± 2.519%(*p* < 0.001; Fig. 3h); and adults 88.54 ± 7.542% versus 19.49 ± 11.43% (*p* < 0.001; Fig. 3l), respectively.

### Characterization of BrdU^+^/EAE-AS^+^ cells in naive neonates, postnates, and adult mice

**Fig. 4:**
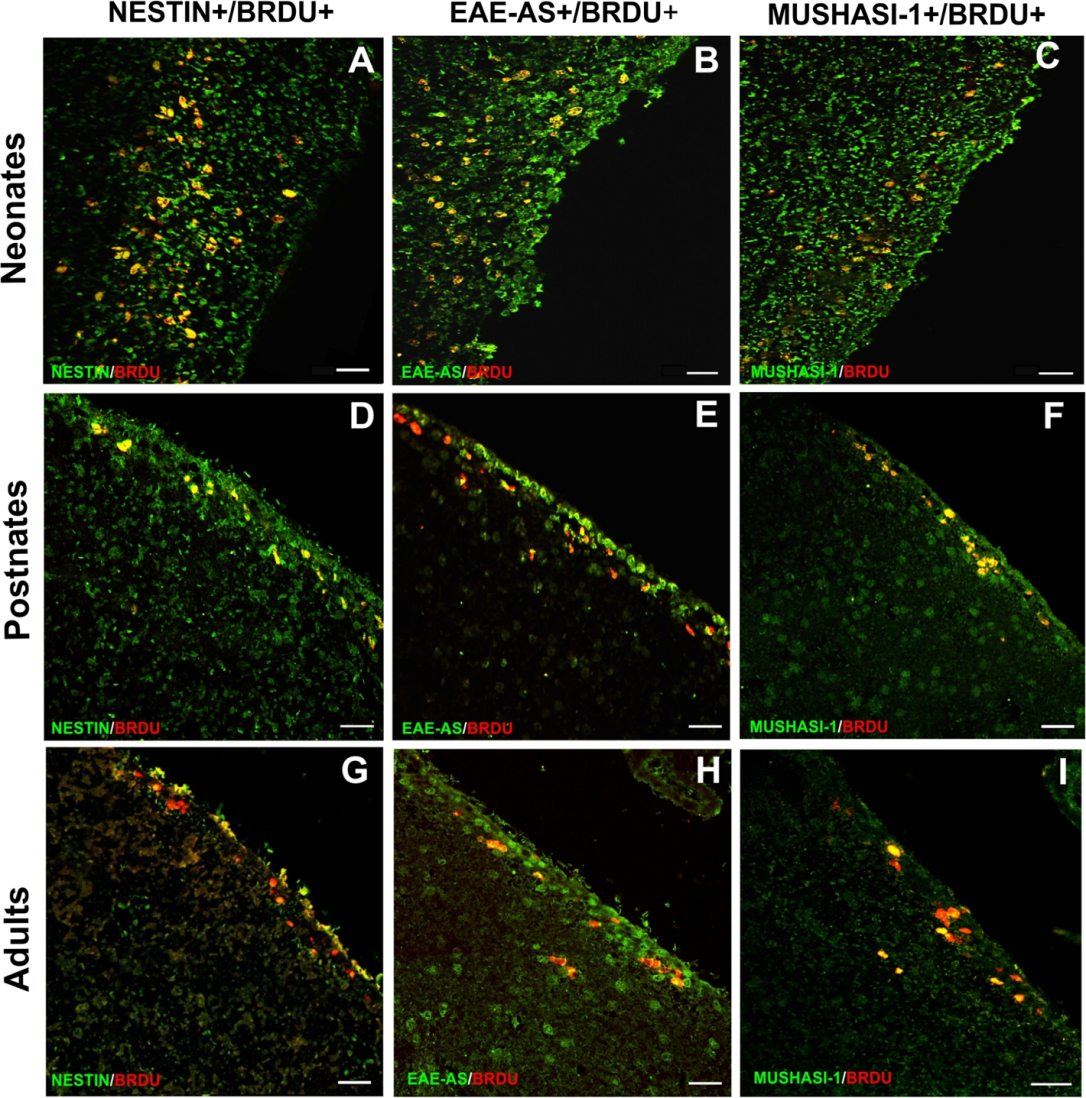
Serial brain sections of developing normal mouse brain co-immunostained with Nestin^+^/BrdU^+^, EAE-AS^+^/BrdU^+^_,_ and Musashi-1^+^/BrdU^+^. Neonates (**a**–**c**). Postnates (**d**–**f**). Adults (**g**–**i**). The majority of progenitor cells (BrdU^+^) that co-stained with EAE-AS are positive for Nestin and Musashi-1. Yellow denotes double positivity. Scale bars=30 μm

In line with the detection of BrdU^+^/EAE-AS^+^ in SVZ that demonstrates a selective binding to this brain location, identification of the double positive cells followed. We selected markers that are well established for NPC characterization in terms of CNS lineage. Nestin and Mushashi-1 markers revealed the following percentages with BrdU colocalization: in neonate naive animals (Fig. 4a–c), Nestin 82.30 ± 7.464%, Musashi-1 78.86 ± 5.851%; in postnates (Fig. 4d–f), Nestin 77.20 ± 9.476%, Musashi-1 75.82 ± 4.822%; and in adults, Nestin 62.13 ± 7.94%, Musashi-1 61.26 ± 6.208% (Fig. 4g–i; not statistically significant).

**Fig. 5:**
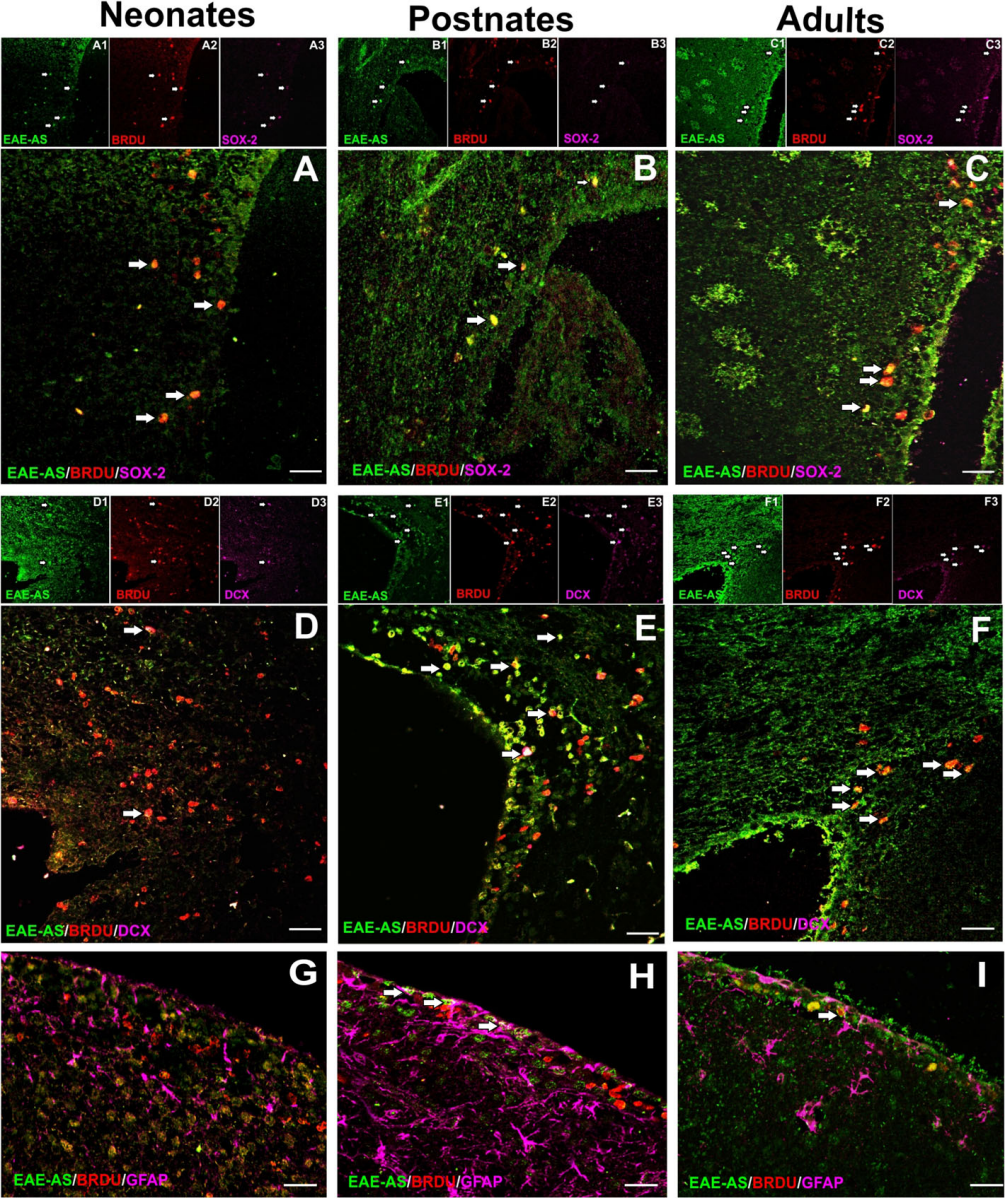
SOX-2, DCX, and GFAP co-labeling with BrdU and EAE-AS in SVZ and periventricular areas. Neonates: SOX-2 (**a**) colocalizes strongly with EAE-AS^+^/BrdU^+^ cells, while DCX (**d**) co-binding is remarkably less. Postnates: SOX-2 (**b**) colocalization with EAE-AS^+^/BrdU^+^ cells diminishes while DCX (**e**) escalates, reaching total colocalization with EAE-AS^+^/BrdU^+^ cells in adult group (**f**). SOX-2 co-binding levels at adults (**c**) are similar to neonate levels. GFAP colocalization levels vary among three age groups. In neonates (**g**) GFAP is hardly co-labeled with EAE-AS^+^/BrdU^+^ cells, whereas in postnates (**h**), a sharp increase is noted. In adults (**i**), co-immunostaining is still detected at lower rates. Arrows define triple positive cells and insets above each photo represent signal of each separate marker. Scale bars=30 μm

Additional NPC markers were used to label the BrdU^+^/EAE-AS^+^ cells. SRY (sex-determining region Y)-box 2 (SOX-2) and doublecortin (DCX) antibodies were applied. Co-labelling with BrdU^+^/EAE-AS^+^ was observed and analyzed: SOX-2 neonates (Fig. 5a) 78.71 ± 8.325%, postnates (Fig. 5b) 45.61 ± 11.78%, and adults (Fig. 5c) 77.45 ± 9.227% (not statistically significant); DCX neonates (Fig. 5d) 13.073 ± 3.840%, postnates (Fig. 5e) 90.83 ± 3.493%, and adults 100.00 ± 0.0% (*p* < 0.001, among the three groups; Fig. 5f).

Another available marker, glial fibrillary acidic protein (GFAP), showed decreased levels of colocalization with BrdU^+^/EAE-AS^+^, especially in neonates (Fig. 5g), 3.33 ± 2.108%. Postnates and adults exhibited higher levels, 68.36 ± 4.362% and 37.38 ± 9.994%, respectively (*p* < 0.01, among the three groups; Fig. 5h, i).

All the above findings verify EAE-AS selective binding to NPCs in SVZ niche and a preference to cells of early neurogenesis stages. Moreover, observations of neural/glial antigen 2 (NG2) marker co-labeling suggest that albeit the existence of double positivity of BrdU^+^ with NG2^+^ in three age groups, only a few cells in the adult group (14.31%) were triple positive for BrdU^+^/EAE-AS^+^/NG2^+^, while no positive cells were observed in neonates and postnates (data not shown). Accumulating evidence challenge the notion that EAE-AS would only target fully differentiated glial cells and thus may support the hypothesis that EAE-AS exerts cross-reactivity with NPCs, major contributors to restoration process during demyelination.

### Immunoreactivity of antisera on neurospheres after antisera incubation

To further assess whether different antisera (NAIVE-AS, CFA-AS, EAE-AS) could elicit apoptosis and/or autophagy, they were added in culture flasks for 48 h in concentration 100 μg/ml. Corresponding markers, Caspase 3 and Beclin 1, were used for staining fixed neurospheres and fixed N2a cells as a negative control. ICC results demonstrated a significantly high expression of Caspase 3 in neurospheres challenged with EAE-AS versus the two other groups (EAE-AS 16.07 ± 1.196%; CFA-AS 10.42 ± 0.5410%; NAIVE-AS 9.554 ± 0.5684%, *p* < 0.001). Expression of Caspase 3 in N2a cells was not significantly different (EAE-AS 2.502 ± 0.3758%; CFA-AS 3.142 ± 0.4196%; NAIVE-AS 3.109 ± 0.3580% *p* > 0.05). When treated with EAE-AS, the comparison of Caspase 3 expression between neurospheres and N2a is highly significant (*p* < 0.0001). Beclin 1 data showed increased expression in neurospheres stimulated with EAE-AS compared to CFA-AS-treated NPCs (EAE-AS 15.42 ± 1.140%; CFA-AS 7.397 ± 0.8568%, *p* < 0.001). Beclin 1 expression in N2a cells was very low (EAE-AS 2.923 ± 0.4089%; CFA-AS 2.772 ± 0.3817%, *p* > 0.05) and comparison between EAE-AS-treated neurospheres and N2a cells is highly significant (*p* < 0.0001).

## Discussion

In the present study, we provide comprehensive results regarding the role of humoral immunity in the induced demyelination occurring in EAE. During EAE, lesions are created after active immunization with the encephalitogenic peptide MOG_35–55_ and autoantibodies produced in the periphery are able to reach the CNS through a disrupted blood brain barrier [22]. Autoantibodies against MOG cannot cause EAE on their own. It is believed that they enhance T cell activity and probably lead to myelin sheath disruption via complement activation [23, 24]. Research of B cells and autoantibodies contributes to the challenging exploration of MS autoantigen(s). In the course of EAE, the resulting inflammatory brain cell damage leads to the release of various antigens which may take part in the immune response. Our results indicate that activation of the immune system towards MOG_35–55_ induces the production of other antibodies apart from the antibodies related directly to this specific antigen. These findings are the first to our knowledge to indicate that such antibodies exhibit specific cross-linking to NPC surface. Several studies on NPC transplantation in EAE mice, as cell regenerative therapy, have proved that NPCs have the ability to migrate into areas of inflammation/demyelination and improve clinical symptoms [17, 25–27]. Notably, it has also been reported that NPCs from SVZ adult mice migrate to disrupted regions in order to promote remyelination (EAE/MS) [6, 28].

The first sign of antigen-specific antibody response in antisera collected from EAE mice was verified by western blotting of the following substrates: NPC lysate, normal mouse spinal cord extract, and recombinant MOG and by immunocytochemistry of MOG-transduced EL4 cells. Our findings showed that EAE-AS recognized the anticipated MOG protein in spinal cord substrate, but in NPC lysate, different protein bands were identified. Moreover, EAE-AS and anti-MOG exerted immunoreactivity towards recombinant MOG and MOG-transduced EL4 cells (MOG positive control cells). Additionally, ICC revealed that EAE-AS exhibited a higher affinity on dissociated NPCs compared to NAIVE-AS. These screening findings were the trigger to further investigate whether EAE-AS exerts cross-reactivity to SVZ-located NPCs and whether this conjugation is unique to a certain NPCs subtype, in terms of CNS lineage (glia and/or neurons).

Numerous anti-myelin antibodies have been recognized in MS pathology, against MBP, PLP, alpha-beta-crystallin, and MOG. During MS course, titres of all the above autoantibodies differ depending on MS stage and progression [13]. MOG 25 kDa is widely known to have a molecular weight of 26–28 kDa when partially glycosylated and has the ability to form 52–56 kDa dimers [21]. Autoantibodies against MOG mediate demyelination in vitro [10] and in experimental models of MS (e.g., EAE) [11], targeting extracellular epitopes of MOG [12]. Studies in MS patients have shown that autoantibodies against MOG recognize linear and conformational epitopes, with stronger antigenicity of the latter [29]. Detectable concentrations of anti-MOG antibodies have been found in both patients’ and healthy people’s sera which disputes the use of such antibodies as MS biomarker [30]. Our data demonstrate that administration of MOG in EAE leads to humoral response with the production of antibodies targeting MOG (28–30 kDa) and antibodies targeting a different set of proteins (around 30 kDa, above 40 kDa, around 45 kDa, and above 60 kDa) found on cells participating in brain lesion restoration (NPCs).

In agreement, we observed a specific binding of circulating antibodies of EAE-AS in the subventricular and periventricular brain areas in neonates, postnates, and adult mice. Our findings revealed significantly increased binding of EAE-AS compared to NAIVE-AS with notably higher levels in the adult group. SVZ cells can give rise to CNS lineage and is an important source of astrocytes and oligodendrocytes (glial cells). SVZ contains four different types of cells: (1) ependymal cells (type E) are in contact with the lumen of the ventricle and are involved in the circulation of CSF; (2) migrating precursor neurons and neuroblasts (type A) expressing PSA-NCAM, Tuj1, and Hu that can migrate through the rostral migratory stream (RMS) to the olfactory bulb; (3) slowly dividing cells (type B) with NPC properties and characteristics of astrocytes. They express Nestin and GFAP and give rise to neuroblasts (type A) or differentiate into precursor cells (type C); and (4) rapidly dividing precursor cells (type C) that express Nestin. The SVZ appears to follow a specific pattern regarding cell proliferation and maturation: Cells of type B (slowly proliferating) to type C (rapidly diving) and then to type A (migrating neuroblasts) [31–33]. Cells of SVZ start to generate astrocytes during the last embryonic days (E19 in rodents). The maturation of astrocytes peaks during postnatal day P0 to P2, whereas mature oligodendrocytes appear later at postnatal day P14 [34, 35]. Therefore, the generation of astrocytes precedes that of oligodendrocytes.

Further insights into EAE-AS selective binding to NPC surface required thorough examination of SVZ brain sections of naive neonatal, postnatal, and adult mice. Significant BrdU^+^/EAE-AS^+^ colocalization was confirmed in all age groups, while BrdU^+^/anti-MOG^+^ co-localization was very limited. The latter observation corroborates the commonly known fact that NPCs in their majority do not express MOG on their surface until differentiated to mature myelinating oligodendrocytes. Hence, these outcomes question the selective binding of autoantibodies (included in EAE-AS) confined to MOG-expressing cells.

In order to determine which lineage-specific NPC type binds with EAE-AS, well-established NPC markers were used in addition to BrdU positivity. Nestin is widely known as a CNS neural/progenitor cell marker and is not expressed in brain regions that host terminally differentiated cells [36, 37]. Cells positive for Nestin exhibit properties of multipotency, self-renewal, and regenerative ability. The onset of neurogenesis (embryonic tissues) is accompanied by Nestin expression with a gradual downregulation as neural differentiation is completed. The expression of Nestin is succeeded by GFAP and neurofilaments [38]. Nestin-positive cells are located in adult CNS areas where progenitor cells are hosted and can migrate in response to tissue damage during regeneration [39].

Another specific marker for early stages of neurogenesis is mouse Musashi-1 (m-Musashi-1), an RNA-binding protein expressed principally in NPCs that differentiate to neuronal and/or glial cell lines, albeit not expressed in mature neurons [40]. During transition from embryonic to early postnatal environment, as CNS develops, m-Musashi-1 can be observed in gradually lower levels [41]. In embryonic life, m-Musashi-1^+^ cells are predominantly located in the ventricular zone (VZ) of the forebrain and spinal cord. In the early postnatal period, m-Musashi-1^+^ cells lie in areas such as SVZ, RMS, olfactory bulb, and in the adult subependymal zone. M-Musashi-1 is highly expressed in embryonic VZ/postnatal SVZ on proliferating undifferentiated stem cells prone to differentiate into neurons, oligodendrocytes, and astrocyte lineages, a fate which is genetically and microenvironmentally regulated. Nestin and Musashi-1 expression patterns in the developing mouse brain are in accordance with our experimental findings. Cells expressing Nestin and m-Musashi-1 are diminishing during the transition from neonates (P3) to adults (3 months old). In parallel, we observed abundance of cells in three age groups that are positive for Nestin^+^/Musashi-1^+^/EAE-AS^+^. These findings lead to the notion that EAE-AS targets cells with the capacity to give rise to neurons and glia.

Sox2, another marker for neural stem cells, is crucial for brain and spinal cord development [42]. In particular, Sox2 protein levels are expressed on neural stem cells, glial progenitors, and developing astrocytes while not expressed on neurons. Sox2 is significantly reduced postnatally and located on SVZ NPCs, on ependymal cells, and on a small fraction of proliferating astrocytes [43]. Cells migrating from the SVZ area lose Sox2 expression as they differentiate into neurons. When astrocytes become fully mature, gene expression is inactivated, whereas upon CNS trauma they are recruited to regenerate tissue lesion and re-express Sox2 [44]. Our work on Sox2 protein levels supports the above reports. Regarding BrdU^+^/EAE-AS^+^ cells colocalization with Sox2, we observed high levels of BrdU^+^/EAE-AS^+^/Sox2^+^ at early postnatal life (P3) and diminished levels postnatally (P17) while in the adult group (3 months old), mild increase of Sox2 co-labeling was noted (*p* > 0.05).

Antisera from mice immunized with MOG were characterized by affinity to NPCs and co-labelling with NPC markers that correspond to multipotency and cell proliferation. In order to explore whether EAE-AS exhibits similar binding affinity to dividing cells of glial or neuronal lineage, we conducted tIF with DCX and GFAP.

Although DCX is a transient protein marker of newborn neuronal cells, it is not expressed on neurons that have been activated after brain injury. During embryogenesis and early stages of postnatal life, expression levels of DCX are medium to low. As the mouse brain evolves, DCX expression levels augment predominantly in the lateral ventricle, dentate gyrus, and olfactory bulb [45]. When full maturation occurs, DCX is observed only in regions of neurogenesis [46]. The current study reveals that colocalization of EAE-AS^+^/BrdU^+^/DCX^+^ alters from the neonatal to the adult stage. Low staining levels with the neuronal marker DCX in P3 animals is indicative of a multipotent stem cell population not yet terminally differentiated. During CNS aging, colocalization of EAE-AS^+^/BrdU^+^/DCX^+^ augments, as the proportion of stem cells decreases and cell fate is determined.

Furthermore, GFAP was applied for astrocyte labeling, although available data demarcate it as a neuronal and oligodendrocyte CNS marker [47, 48]. GFAP protein is observed at E12 in mouse brain with escalating expression as development progresses [49]. Our results regarding GFAP expression levels are in parallel with existing reports while colabelling with BrdU^+^/EAE-AS^+^ cells varies. In neonates, colocalization is hardly observed, while in postnates and adults, there is a remarkable increase. One possible explanation relies on the fact that although progenitor cells are observed in three age groups, NPCs prevail mainly in neonates, whereas differentiating cells (GFAP^+^) are increased in postnates and adults.

NG2 is expressed mainly on precursor cells of glial lineage (oligodendrocytes and astrocytes). In developing mouse brain and spinal cord, NG2^+^ cells are found in early embryonic stages (E13) reaching a peak at P8–P12 and progressively decreasing until adulthood [50]. Our findings are in an accordance with NG2 distribution in normal brain sections while minimum NG2 co-expression with BrdU^+^/EAE-AS^+^ binding was found and specifically only in the adult group. Moreover, NG2^+^ cells may participate in remyelination process as they have been observed in MS demyelinated regions [51, 52]. It is still unclear whether these NG2^+^ cells exert a true OPC activity.

Another interesting question would be whether these antisera are pathogenic or not to NPCs. To our knowledge, antibodies act with specific mechanisms: direct apoptotic activity, antibody-dependent cellular cytotoxicity (ADCC) by immune effector cells, and complement-dependent cytotoxicity (CDC) [53]. In order to investigate the potential of antisera to induce apoptosis and/or autophagy on NPCs, we performed in vitro incubation of the cells with NAIVE-AS, CFA-AS, and EAE-AS for 48 h and stained with Caspase 3 (key apoptotic molecule) and Beclin 1 (key autophagy mediator). The latter molecule upregulates autophagy whereas Caspase 3 has the ability to inactivate Beclin 1 by cleavage and render cells prone to apoptosis [54, 55]. Our data demonstrate an increased expression of both mediators in NPCs treated with EAE-AS opposed to CFA-AS- and NAIVE-AS-treated NPCs. These findings suggest that antisera from MOG_35–55_-immunized mice may provoke the mechanism of apoptosis and autophagy.

## Conclusions

The current experimental research demonstrates that antisera derived from rodents immunized with MOG_35–55_ may exert selective binding to SVZ stem cells, which are main effectors of remyelination through migration and proliferation into inflammatory demyelinating foci. EAE-AS cross-reactivity to NPCs and their progeny may dictate that endogenous NPCs are influenced phenotypically and functionally by EAE immune responses. Therefore, NPC-driven tissue remyelination is either impeded or rendered ineffective.

Data so far reported state that MOG is not expressed on NPC surface and is only found on mature myelinating oligodendrocytes. Although serum antibodies in MS patients [56] and EAE animals [57] have been extensively investigated, their affinity to SVZ area and cross-linking to NPCs have not been addressed. In vitro analysis showed that EAE-AS has an impact on NPCs through activation of the apoptotic pathway. This is the first report, to our knowledge, that autoantibodies in EAE may differentially target specific precursor cells of SVZ on an age-dependent basis. Whether in MS a respective humoral response is activated against neural precursor cells remains to be investigated. Interestingly enough, data from multicenter clinical trials with the use of anti-B cell monoclonal antibodies, such as anti-CD20 in MS [58, 59], provide clear evidence on the beneficial effect of this treatment in controlling disease activity and disability progression. It would be of importance to investigate whether the same monoclonal antibodies may have any impact on NPC kinetics in the ongoing demyelinating process. Further studies of interactions between autoantibodies and endogenous NPCs, regarding their differentiation/migration and activation of ADCC/CDC mechanisms, are crucial for understanding the tight balance of homeostasis in rodent and human CNS.

## Additional files

Additional file 1: Methods. Experimental groups, EAE induction and evaluation, Cell culture protocol, Preparation of samples for SDS-PAGE. (RTF 3.45 kb)

Additional file 2: Figure S1. Evidence of the production of anti-MOG-immunoglobulins within antisera when mice were inoculated with MOG peptide. (**A**) Purified EAE-AS (IgG from EAE-AS) and unpurified EAE-AS identified bands of the same molecular weight on NPCs substrate. Western blot of recombinant MOG as SDS-PAGE substrate (**B**) and ICC of EL4-MOG cells (**C**) revealed the real existence of anti-MOG-immunoglobulins within EAE-AS. One band was yielded (above 20kDA) when recombinant MOG was probed with EAE-AS and anti-MOG (positive control) (**B**). EAE-AS and anti-MOG showed high levels of binding on EL4-MOG cells (DAB staining), whereas NAIVE-AS did not bind (**C**). Magnification=40X, Scale=100μm. (JPEG 344 kb)

## Abbreviations

ADCC: Antibody-dependent cellular cytotoxicity
BCR: B- cell receptor
bFGF: Basic fibroblast growth factor
BrdU: 5-bromo-2′-deoxyuridine
CDC: Complement-dependent cytotoxicity
CFA: Complete Freund’s adjuvant
CNS: Central nervous system
CSF: Cerebrospinal fluid
DAB: 3,3′ diaminobenzidine-tetra-hydrochloride
DAPI: 4′,6-diamidino-2-phenylindole
DCX: Doublecortin
EAE: Experimental autoimmune encephalomyelitis
ECL: Enhanced chemiluminescence
EGF: Epidermal growth factor
GFAP: Glial fibrillary acidic protein
MBP: Myelin basic protein
MOG: Myelin oligodendrocyte glycoprotein
MS: Multiple sclerosis
MT: Mycobacterium tuberculosis
NG2: Neural/glial antigen 2
NPCs: Neural precursor cells
OPCs: Oligodendrocyte precursor cells
PLP: Myelin proteolipid protein
SEM: Standard error of the mean
SGZ: Subgranular zone
SOX-2: Sex-determining region Y-box 2
SVZ: Subventricular zone

## References

[1] Qin Y, Duquette P, Zhang Y, Talbot P, Poole R, Antel J (1998). Clonal expansion and somatic hypermutation of V(H) genes of B cells from cerebrospinal fluid in multiple sclerosis. J Clin Investig.

[2] Obermeier B, Mentele R, Malotka J, Kellermann J, Kumpfel T, Wekerle H, Lottspeich F, Hohlfeld R, Dornmair K (2008). Matching of oligoclonal immunoglobulin transcriptomes and proteomes of cerebrospinal fluid in multiple sclerosis. Nat Med.

[3] Conover JC, Notti RQ (2007). The neural stem cell niche. Cell Tissue Res.

[4] Alvarez-Buylla A, Seri B, Doetsch F (2002). Identification of neural stem cells in the adult vertebrate brain. Brain Res Bull.

[5] Picard-Riera N, Nait-Oumesmar B, Baron-Van Evercooren A (2004). Endogenous adult neural stem cells: limits and potential to repair the injured central nervous system. J Neurosci Res.

[6] Martino G, Pluchino S (2006). The therapeutic potential of neural stem cells. Nat Rev Neurosci.

[7] Keirstead HS (2001). Stem cell transplantation into the central nervous system and the control of differentiation. J Neurosci Res.

[8] Fainstein N, Vaknin I, Einstein O, Zisman P, Sasson SZB, Baniyash M, Ben-Hur T (2008). Neural precursor cells inhibit multiple inflammatory signals. Mol Cell Neurosci.

[9] Pagany M, Jagodic M, Bourquin C, Olsson T, Linington C (2003). Genetic variation in myelin oligodendrocyte glycoprotein expression and susceptibility to experimental autoimmune encephalomyelitis. J Neuroimmunol.

[10] della Gaspera B, Pham-Dinh D, Roussel G, Nussbaum J-L, Dautigny A (1998). Membrane topology of the myelin/oligodendrocyte glycoprotein. Eur J Biochem.

[11] Lassmann H, Brunner C, Bradl M, Linington C (1988). Experimental allergic encephalomyelitis: the balance between encephalitogenic T lymphocytes and demyelinating antibodies determines size and structure of demyelinated lesions. Acta Neuropathol.

[12] Adelmann M, Wood J, Benzel I, Fiori P, Lassmann H, Matthieu JM, Gardinier MV, Dornmair K, Linington C (1995). The N-terminal domain of the myelin oligodendrocyte glycoprotein (MOG) induces acute demyelinating experimental autoimmune encephalomyelitis in the Lewis rat. J Neuroimmunol.

[13] Ziemssen T, Ziemssen F (2005). The role of the humoral immune system in multiple sclerosis (MS) and its animal model experimental autoimmune encephalomyelitis (EAE). Autoimmun Rev.

[14] Theotokis P, Kleopa KA, Touloumi O, Lagoudaki R, Lourbopoulos A, Nousiopoulou E, Kesidou E, Poulatsidou K-N, Dardiotis E, Hadjigeorgiou G (2015). Connexin43 and connexin47 alterations after neural precursor cells transplantation in experimental autoimmune encephalomyelitis. Glia.

[15] Theotokis P, Lourbopoulos A, Touloumi O, Lagoudaki R, Kofidou E, Nousiopoulou E, Poulatsidou K-N, Kesidou E, Tascos N, Spandou E, Grigoriadis N (2012). Time course and spatial profile of Nogo-A expression in experimental autoimmune encephalomyelitis in C57BL/6 mice. J Neuropathol Exp Neurol.

[16] Lehner B, Sandner B, Marschallinger J, Lehner C, Furtner T, Couillard-Despres S, Rivera FJ, Brockhoff G, Bauer HC, Weidner N, Aigner L (2011). The dark side of BrdU in neural stem cell biology: detrimental effects on cell cycle, differentiation and survival. Cell Tissue Res.

[17] Einstein O, Grigoriadis N, Mizrachi-Kol R, Reinhartz E, Polyzoidou E, Lavon I, Milonas I, Karussis D, Abramsky O, Ben-Hur T (2006). Transplanted neural precursor cells reduce brain inflammation to attenuate chronic experimental autoimmune encephalomyelitis. Exp Neurol.

[18] Giannakopoulou A, Grigoriadis N, Polyzoidou E, Lourbopoulos A, Michaloudi E, Papadopoulos GC (2011). Time-dependent fate of transplanted neural precursor cells in experimental autoimmune encephalomyelitis mice. Exp Neurol.

[19] Pollinger B, Krishnamoorthy G, Berer K, Lassmann H, Bosl MR, Dunn R, Domingues HS, Holz A, Kurschus FC, Wekerle H (2009). Spontaneous relapsing-remitting EAE in the SJL/J mouse: MOG-reactive transgenic T cells recruit endogenous MOG-specific B cells. J Exp Med.

[20] Koukouli F, Paspaltsis I, Salta E, Xanthopoulos K, Koini EN, Calogeropoulou T, Sklaviadis T (2012). Inhibition of PrP(Sc) formation in scrapie infected N2a cells by 5,7,8-trimethyl-3,4-dihydro-2H-1,4-benzoxazine derivatives. Prion.

[21] Amiguet P, Gardinier MV, Zanetta J-P, Matthieu J-M (1992). Purification and partial structural and functional characterization of mouse myelin/oligodendrocyte glycoprotein. J Neurochem.

[22] Goverman J (2009). Autoimmune T cell responses in the central nervous system. Nat Rev Immunol.

[23] von Budingen HC, Bar-Or A, Zamvil SS (2011). B cells in multiple sclerosis: connecting the dots. Curr Opin Immunol.

[24] Berer K, Wekerle H, Krishnamoorthy G (2011). B cells in spontaneous autoimmune diseases of the central nervous system. Mol Immunol.

[25] Ben-Hur T, Einstein O, Mizrachi-Kol R, Ben-Menachem O, Reinhartz E, Karussis D, Abramsky O (2003). Transplanted multipotential neural precursor cells migrate into the inflamed white matter in response to experimental autoimmune encephalomyelitis. Glia.

[26] Ben-Hur T, Einstein O, Bulte JW (2005). Stem cell therapy for myelin diseases. Curr Drug Targets.

[27] Einstein O, Friedman-Levi Y, Grigoriadis N, Ben-Hur T (2009). Transplanted neural precursors enhance host brain-derived myelin regeneration. J Neurosci.

[28] Calza L, Giardino L, Pozza M, Bettelli C, Micera A, Aloe L (1998). Proliferation and phenotype regulation in the subventricular zone during experimental allergic encephalomyelitis: in vivo evidence of a role for nerve growth factor. Proc Natl Acad Sci U S A.

[29] Brehm U, Piddlesden SJ, Gardinier MV, Linington C (1999). Epitope specificity of demyelinating monoclonal autoantibodies directed against the human myelin oligodendrocyte glycoprotein (MOG). J Neuroimmunol.

[30] Iglesias A, Bauer J, Litzenburger T, Schubart A, Linington C (2001). T- and B-cell responses to myelin oligodendrocyte glycoprotein in experimental autoimmune encephalomyelitis and multiple sclerosis. Glia.

[31] Doetsch F, Garcia-Verdugo JM, Alvarez-Buylla A (1997). Cellular composition and three-dimensional organization of the subventricular germinal zone in the adult mammalian brain. J Neurosci.

[32] Lois C, Alvarez-Buylla A (1993). Proliferating subventricular zone cells in the adult mammalian forebrain can differentiate into neurons and glia. Proc Natl Acad Sci.

[33] Doetsch F, Caillé I, Lim DA, García-Verdugo JM, Alvarez-Buylla A (1999). Subventricular zone astrocytes are neural stem cells in the adult mammalian brain. Cell.

[34] Parnavelas JG (1999). Glial cell lineages in the rat cerebral cortex. Exp Neurol.

[35] Levison SW, Goldman JE (1993). Both oligodendrocytes and astrocytes develop from progenitors in the subventricular zone of postnatal rat forebrain. Neuron.

[36] Reynolds B, Weiss S (1992). Generation of neurons and astrocytes from isolated cells of the adult mammalian central nervous system. Science.

[37] Gage FH (2000). Mammalian neural stem cells. Science.

[38] Lendahl U, Zimmerman LB, McKay RD (1990). CNS stem cells express a new class of intermediate filament protein. Cell.

[39] Wiese C, Rolletschek A, Kania G, Blyszczuk P, Tarasov KV, Tarasova Y, Wersto RP, Boheler KR, Wobus AM: Nestin expression? A property of multi-lineage progenitor cells? *CMLS,* Cell Mol Life Sci 2004, 61**:**2510-2522. 10.1007/s00018-004-4144-6 PMC11924557 15526158

[40] S-i S, Nakamura Y, Takano H, Noda T, Okano H (1998). Mouse musashi-1, a RNA-binding protein enriched in mammalian neural precursor cells. Neurosci Res.

[41] Sakakibara S-i, Okano H: 1004 Expression of neural RNA-binding proteins in the postnatal CNS: implications of their roles in neuronal and glial cells 1**.** Neurosci Res 1997, 28**:**S117. 10.1523/JNEUROSCI.17-21-08300.1997 PMC6573750 9334405

[42] Ferri ALM (2004). Sox2 deficiency causes neurodegeneration and impaired neurogenesis in the adult mouse brain. Development.

[43] Ellis P, Fagan BM, Magness ST, Hutton S, Taranova O, Hayashi S, McMahon A, Rao M, Pevny L (2004). SOX2, a persistent marker for multipotential neural stem cells derived from embryonic stem cells, the embryo or the adult. Dev Neurosci.

[44] Bani-Yaghoub M, Tremblay RG, Lei JX, Zhang D, Zurakowski B, Sandhu JK, Smith B, Ribecco-Lutkiewicz M, Kennedy J, Walker PR, Sikorska M (2006). Role of Sox2 in the development of the mouse neocortex. Dev Biol.

[45] Walker TL, Yasuda T, Adams DJ, Bartlett PF (2007). The doublecortin-expressing population in the developing and adult brain contains multipotential precursors in addition to neuronal-lineage cells. J Neurosci.

[46] Brown JP, Couillard-Després S, Cooper-Kuhn CM, Winkler J, Aigner L, Kuhn HG (2003). Transient expression of doublecortin during adult neurogenesis. J Comp Neurol.

[47] Casper KB, McCarthy KD (2006). GFAP-positive progenitor cells produce neurons and oligodendrocytes throughout the CNS. Mol Cell Neurosci.

[48] Kamphuis W, Mamber C, Moeton M, Kooijman L, Sluijs JA, Jansen AHP, Verveer M, de Groot LR, Smith VD, Rangarajan S (2012). GFAP isoforms in adult mouse brain with a focus on neurogenic astrocytes and reactive astrogliosis in mouse models of Alzheimer disease. PLoS One.

[49] Mamber C, Kamphuis W, Haring NL, Peprah N, Middeldorp J, Hol EM (2012). GFAPδ expression in glia of the developmental and adolescent mouse brain. PLoS One.

[50] Niehaus A, Shi J, Grzenkowski M, Diers-Fenger M, Archelos J, Hartung HP, Toyka K, Bruck W, Trotter J (2000). Patients with active relapsing-remitting multiple sclerosis synthesize antibodies recognizing oligodendrocyte progenitor cell surface protein: implications for remyelination. Ann Neurol.

[51] Chang A, Nishiyama A, Peterson J, Prineas J, Trapp BD (2000). NG2-positive oligodendrocyte progenitor cells in adult human brain and multiple sclerosis lesions. J Neurosci.

[52] Karram K, Chatterjee N, Trotter J (2005). NG2-expressing cells in the nervous system: role of the proteoglycan in migration and glial-neuron interaction. J Anat.

[53] Weiner GJ (2015). Building better monoclonal antibody-based therapeutics. Nat Rev Cancer.

[54] Kesidou E, Lagoudaki R, Touloumi O, Poulatsidou KN, Simeonidou C (2013). Autophagy and neurodegenerative disorders. Neural Regen Res.

[55] Marino G, Niso-Santano M, Baehrecke EH, Kroemer G (2014). Self-consumption: the interplay of autophagy and apoptosis. Nat Rev Mol Cell Biol.

[56] Reindl M (1999). Antibodies against the myelin oligodendrocyte glycoprotein and the myelin basic protein in multiple sclerosis and other neurological diseases: a comparative study. Brain.

[57] Bansal P, Khan T, Bussmeyer U, Challa DK, Swiercz R, Velmurugan R, Ober RJ, Ward ES (2013). The encephalitogenic, human myelin oligodendrocyte glycoprotein-induced antibody repertoire is directed toward multiple epitopes in C57BL/6-immunized mice. J Immunol.

[58] Cross AH, Naismith RT (2014). Established and novel disease-modifying treatments in multiple sclerosis. J Intern Med.

[59] Moreno Torres I, Garcia-Merino A (2017). Anti-CD20 monoclonal antibodies in multiple sclerosis. Expert Rev Neurother.

