# Supplementary material for: Humoral response in experimental autoimmune encephalomyelitis targets neural precursor cells in the central nervous system of naive rodents
Source: J Neuroinflammation. 2017 Nov 21;14:227. doi: 10.1186/s12974-017-0995-2 (PMC5697419; doi:10.1186/s12974-017-0995-2)
Supplement: Supplementary file 1 — Methods. Experimental groups, EAE induction and evaluation, Cell culture protocol, Preparation of samples for SDS-PAGE. (RTF 3.45 kb) [file 12974_2017_995_MOESM1_ESM.rtf]

Supplementary material
Additional file 1
Methods

Experimental groups, EAE induction and evaluation
On day 0, mice were inoculated subcutaneously at the para-lumbar region with 300ìg MOG35-55 (kindly provided by Dr. John Matsoukas, Department of Chemistry, University of Patras) in 200ìl of solution consisting of 100ìl CFA [Incomplete Freund's adjuvant (IFA; Sigma-Aldrich GmbH, Germany) supplemented with 4mg/ml mycobacterium tuberculosis H37RA (MT; Thermo Fischer Scientific, Haverhill, MA, USA)] and 100ìl PBS (phosphate buffer saline). The same administration procedure was repeated on day 7 of the experiment. In the CFA group, mice were administered two CFA injections, on day 0 and day 7, without the addition of antigenic MOG35-55 peptide. Furthermore, on day 0 animals of EAE and CFA group received intraperitoneally (i.p.) 400ng pertussis toxin (Sigma-Aldrich GmbH, Germany)in 0.5ml PBS whereas on day 2 they received 200ng. Furthermore, rodents of EAE, CFA and naive adult group were daily examined, weighed and clinically evaluated for EAE signs using a 6-grade scale: 0, asymptomatic; 1, tail tonicity partial loss; 2, tail paralysis; 3, difficulty to return to upright position from supine; 4, hind limb paralysis; 5, forelimb paresis; and 6, death due to EAE.

Cell culture protocol 
Briefly, NPCs were isolated from cerebral hemispheres and enzymatical (trypsin/ Sigma-Aldrich GmbH, Germany) and mechanical dissociation (pipette syringe) followed. Cells were resuspended in DMEM/F12 medium ( Thermo Fischer Scientific, Haverhill, MA, USA) containing 1mM sodium-pyruvate (Sigma-Aldrich GmbH, Germany), 0,05% bovine serum albumin (BSA; Sigma-Aldrich, GmbH Germany), 10ng/ml D-biotin (Sigma-Aldrich, GmbH, Germany ), 2mM L-glutamine (Thermo Fischer Scientific, Haverhill, MA, USA), 25ìg/ml gentamicin (Sigma-Aldrich GmbH, Germany) and N-2 supplement (Invitrogen) and were plated in T75 (Corning, NY, USA) uncoated culture flasks.

For EL4-MOG, mouse Mog cDNA was cloned into specific vector and transformed into GP+E-86 cell line and transduced the mouse EL4 T-cell lymphoma line. EL4-MOG were cultured in T-75 flasks (Corning, NY, USA) with RPMI-1640 medium supplemented with 10% FBS, 2mM L-glutamine and 1% Penicillin-Streptomycin (Sigma-Aldrich GmbH, Germany). EL4-MOG were cultured until confluent under constant conditions of 5% CO2 and 37ï C.

Preparation of samples for SDS-PAGE
Total protein lysate was isolated from NPC neurospheres and from naive adult mouse spinal cord. Cells were prepared on ice with lysis buffer consisting of 25mM Tris pH 7.4, 150mM NaCl, 0,1%SDS, 1mM DTT, 1% Triton X-100 and protease inhibitors (1mM PMSF, 1ìg/ml leupeptin, 1ìg/ml aprotinin) and then centrifuged at 10400g for 30 min. Spinal cord was homogenized in lysis buffer consisting of 10mM Hepes pH 7.4, 10mM KCL, 0.1mM EDTA, 0.1mM EGTA, 1mM DTT and a mixture of protease inhibitors. Protein concentration of collected supernatant from both NPCs and spinal cord was determined by DC protein assay kit (Biorad, Hercules, California, USA).
