# Supplementary figures and images for: Humoral response in experimental autoimmune encephalomyelitis targets neural precursor cells in the central nervous system of naive rodents
Source: J Neuroinflammation. 2017 Nov 21;14:227. doi: 10.1186/s12974-017-0995-2 (PMC5697419; doi:10.1186/s12974-017-0995-2)

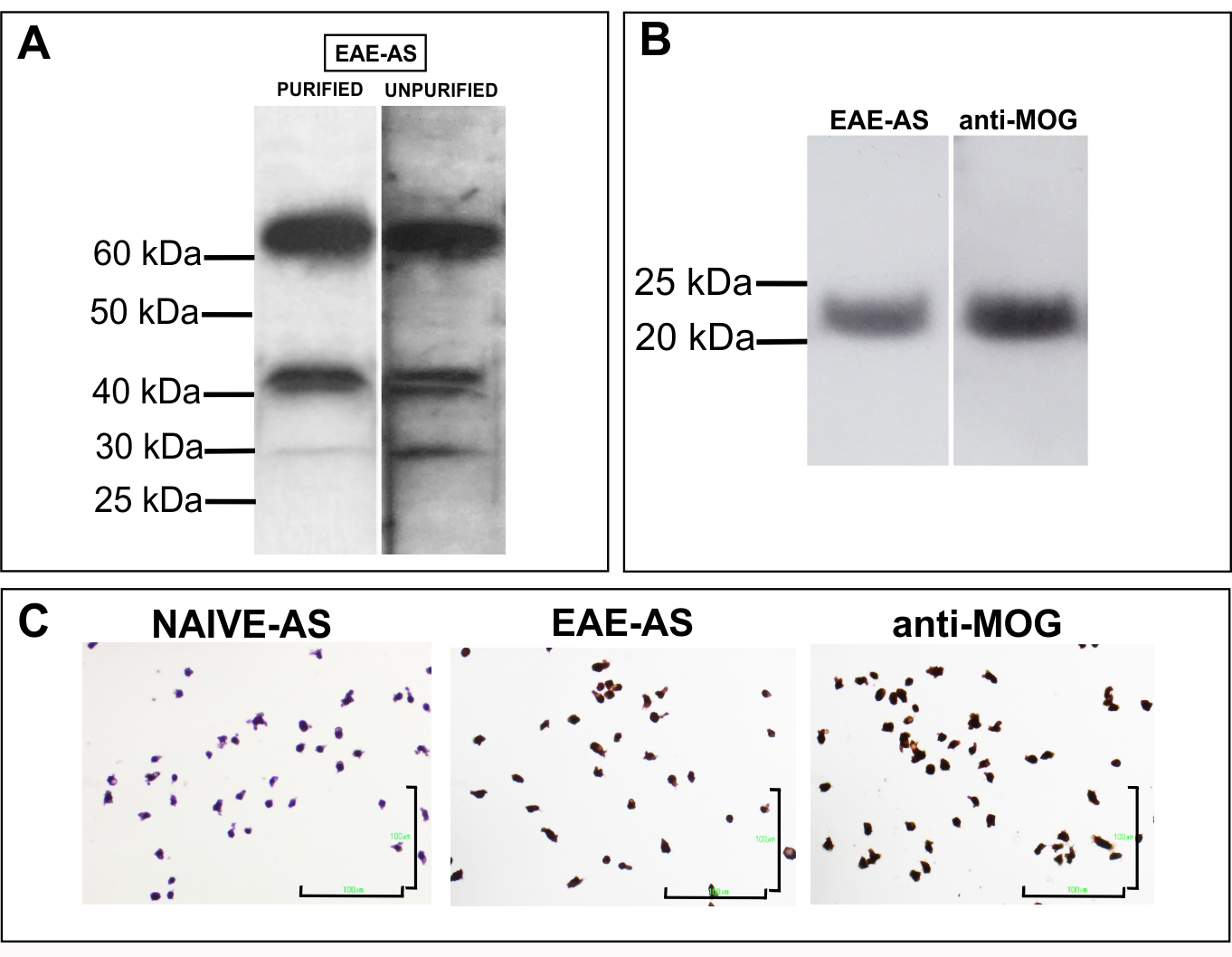

Supplement: Supplementary file 2 — Evidence of the production of anti-MOG-immunoglobulins within antisera when mice were inoculated with MOG peptide. (A) Purified EAE-AS (IgG from EAE-AS) and unpurified EAE-AS identified bands of the same molecular weight on NPCs substrate. Western blot of recombinant MOG as SDS-PAGE substrate (B) and ICC of EL4-MOG cells (C) revealed the real existence of anti-MOG-immunoglobulins within EAE-AS. One band was yielded (above 20kDA) when recombinant MOG was probed with EAE-AS and anti-MOG (positive control) (B). EAE-AS and anti-MOG showed high levels of binding on EL4-MOG cells (DAB staining), whereas NAIVE-AS did not bind (C). Magnification=40X, Scale=100μm. (JPEG 344 kb) [file 12974_2017_995_MOESM2_ESM.jpg]
